# Benefits of social vs. non-social feedback on learning and generosity. Results from the Tipping Game

**DOI:** 10.3389/fpsyg.2014.01154

**Published:** 2014-10-09

**Authors:** Matteo Colombo, Aistis Stankevicius, Peggy Seriès

**Affiliations:** ^1^Tilburg Center for Logic, General Ethics, and Philosophy of Science, Tilburg University, TilburgNetherlands; ^2^Institute for Adaptive and Neural Computation, University of Edinburgh, EdinburghUK

**Keywords:** social/non-social feedback, facial expressions, social norms, tipping behavior, associative learning

## Abstract

Although much work has recently been directed at understanding social decision-making, relatively little is known about how different types of feedback impact adaptive changes in social behavior. To address this issue quantitatively, we designed a novel associative learning task called the “Tipping Game,” in which participants had to learn a social norm of tipping in restaurants. Participants were found to make more generous decisions from feedback in the form of facial expressions, in comparison to feedback in the form of symbols such as ticks and crosses. Furthermore, more participants displayed learning in the condition where they received social feedback than participants in the non-social condition. Modeling results showed that the pattern of performance displayed by participants receiving social feedback could be explained by a lower sensitivity to economic costs.

## INTRODUCTION

Several behavioral, neurobiological and theoretical studies have shown that social norm compliance, and more generally adaptive changes in social behavior, often require the effective use and weighing of different types of information, including expected economic costs and benefits, the potential impact of our behavior on the welfare of others and our own reputation, as well as feedback information (Bicchieri, 2006; Adolphs, 2009; Frith and Frith, 2012). Relatively little attention has been paid to how different types of feedback (or reward) may impact the way social norms are learned. The present study addresses this issue with behavioral and modeling results from a novel associative learning task called the “Tipping Game.” We take the example of tipping and ask: how do social feedback in the form of facial expressions, as opposed to non-social feedback in the form of such conventional signs as ticks and crosses, affect the way participants learn a social norm of tipping?

Recent findings indicate that people’s decision-making is often biased by social stimuli. For example, images of a pair of eyes can significantly increase pro-social behavior in laboratory conditions as well as in real-world contexts (Haley and Fessler, 2005; Bateson et al., 2006; Rigdon et al., 2009; Ernest-Jones et al., 2011). Furthermore, decision-making can be systematically biased by facial emotional expressions used as predictors of monetary reward (Averbeck and Duchaine, 2009; Evans et al., 2011; Shore and Heerey, 2011). Facial expressions of happiness elicit approaching behavior, whereas angry faces elicit avoidance (Seidel et al., 2010; for a review see Blair, 2003). Because they can function as signals to others, eliciting specific behavioral responses, emotional facial expressions play a major role in socialization practices that help individuals to adapt to the norms and values of their culture (Keltner and Haidt, 1999; Frith, 2009).

Despite this body of findings, the literature does not provide an unambiguous answer to the question of how learning performance is affected by social stimuli in comparison to different types of non-social stimuli used as feedback about previous decisions in a learning task (Ruff and Fehr, 2014). Consistent with the view that social reinforcement is a powerful facilitator of human learning (Zajonc, 1965; Bandura, 1977), one recent study using a feedback-guided item-category association task found that learning performance in control groups was improved when social (smiling or angry faces) instead of non-social (green or red lights) reinforcement was used (Hurlemann et al., 2010).

However, the paradigm used in this study did not distinguish between two conditions in which social-facilitative effects on learning performance have been observed: first, a condition characterized by the mere presence of others (Allport, 1920); and second, a condition where others provide reinforcing feedback (Zajonc, 1965). In the task used by Hurlemann et al. (2010), faces were present onscreen throughout each trial, changing from a neutral to a happy expression for correct responses or angry for incorrect responses. So, this study could not identify the specific effect of social feedback on learning.

Consistent with the assumption oft made in economics and psychology that optimal decisions and learning are based on an assessment of the evidence that is unbiased by the social or non-social nature of the evidence itself (Becker, 1976; Oaksford and Chater, 2007), Lin et al. (2012a) found that, instead of boosting learning performance, social reward (smiling or angry faces) made learning slower, and generally less effective, in comparison to non-social reward such as money.

It should be noted that Hurlemann et al. (2010) and Lin et al. (2012a) were examining fundamentally different questions, which may explain the difference in their results. In Hurlemann et al.’s (2010) study, participants used feedback to learn the category membership of an abstract string of numbers, whereas in Lin et al.’s (2012a) study participants played an instrumental learning task where they had to learn to select the slot machine associated with the highest probability of a positive valenced outcome. So, depending on the task, social stimuli may have different, sometimes opposite, effects on learning performance.

In particular, it remains controversial whether participants in an associative learning task receiving feedback in the form of facial expressions learn a social norm more effectively than participants who are provided with non-social, cognitive feedback.

Our study contributes to previous literature by examining more closely the relative impact of social (happy and angry faces) and non-social feedback (tick and cross marks) on learning, and by testing the hypothesis that social feedback leads to more generous behavior, in the context of the Tipping Game. This task tapped into a basic mechanism underlying the ontogeny of social cognition (Reeb-Sutherland et al., 2012), while allowing us to examine the effects of social, as opposed to non-social, feedback on learning and decision-making.

The Tipping Game shares several features with other reinforcement learning tasks, and so the associated modeling framework can be used to quantitatively characterize the behavioral results of both healthy young people—as in our study—as well as clinical and neurological patients (Lin et al., 2012b). In the present study, modeling results helped to disentangle how information carried by specific types of feedback stimuli may interact with economic interest when people are learning a social norm. The originality of the Tipping Game is the social context and social feedback that it involves. These contribute to the higher ecological validity and naturalness of our task, which distinguish it from the ones previously used in studies using facial expressions as predictors of monetary reward (e.g., Averbeck and Duchaine, 2009; Hurlemann et al., 2010; on the importance of the ecological validity in these types of tasks, see Lin et al., 2012b, p. 7).

## EXPERIMENT 1

### METHODS

#### Participants

Forty participants (23 males) made decisions in the Tipping Game. The participants were students in the University of Edinburgh, signed informed consent approved by the University of Edinburgh Ethics Committee, and were compensated with £6/h for taking part in the experiment. While some of the participants were international students, they all lived in the UK for 1 year or more.

#### Design and procedure

The Tipping Game was implemented in Matlab, using the Psychophysics Toolbox 3 extensions (Brainard, 1997; Pelli, 1997; Kleiner et al., 2007), and was run on computers in sound-deadened booths.

Initially, participants filled out five questionnaires: the “Empathy Quotient” (EQ) questionnaire (Baron-Cohen and Wheelwright, 2004), one version of the “Reading the Mind in the Eyes” test (Baron-Cohen et al., 1997), the “Self Report Altruism” questionnaire (Rushton et al., 1981), the “Sensitivity to Punishment and Sensitivity to Reward Questionnaire” (SPSRQ; Torrubia et al., 2001), and the “Behavioral Inhibition/Approach” (BIS/BAS) questionnaire (Carver and White, 1994). These questionnaires measured respectively the level of empathy, mentalizing, altruism, and punishment and reward sensitivity of the participants.

After the questionnaires were completed, instructions were given about the Tipping Game and its goals. Participants were informed that they would take part in a computer-based task where they had to imagine to be diners at restaurants in some unfamiliar country. In each trial of the task, they would be presented firstly with information regarding the quality of the service received at a restaurant (i.e., either good or bad service). Then, the bill to be paid would be disclosed. Finally, participants should decide which amount to tip in that restaurant taking into account the quality of the service. After each decision, either positive or negative feedback would be revealed, indicating that the tip was either higher or lower than expected. Whether positive or negative feedback was revealed depended stochastically on the underlying social norm and on the pair *service quality* – *amount tipped* (see **Table 1**). Feedback information received after a decision could be used by participants to learn how much they were expected to tip.

**Table 1:** Feedback structure in Experiment 1.

Probability of positive feedback (tip < norm/tip ≥ norm)	Block 1 (norm 23%)	Block 2 (norm 50%)	Block 3 (norm 23%)
Good service	20/80	20/80	35/65
Bad service	30/70	30/70	40/60

*Probability of obtaining a positive feedback as a function of the action that was made (tip < norm/tip ≥ norm), and in the situation where the service was good (first line) or bad (second line).*

The task consisted of three blocks, each one of which comprised forty trials. A trial corresponded to one tipping interaction of the sort just described. At the beginning of each block, participants were endowed with 1,100 monetary units (mu), with which they had to pay for restaurant bills and any tip they decided to give. It was made explicit to the participants that the mu they were using were fictional, and that no actual person would benefit from their tips. All participants knew that their goal was to learn a social norm of tipping so as to display adaptive behavior in the social situation they were facing, while saving as much mu as possible.

To motivate participants to pursue this goal, they were told at the beginning of the task that the best overall performance would be rewarded with £20, and that their overall performance would be measured as a function of both how well the social norm was learned and how much mu was saved across the whole task. Participants were also informed that some manipulation could take place across blocks so that they should treat each block as a novel kind of situation (i.e., a different unfamiliar country).

Two types of manipulations took place. First, the underlying social norm of tipping changed between blocks. Second, the reliability of the reward feedback changed across blocks, with feedback being more random in the third block than in the previous blocks (**Table 1**).

In one condition (Social Condition), 20 participants (seven females) received feedback in the form of a happy or an angry face. Pictures of facial expressions were in greyscale and selected from the Japanese Female Facial Expression (JAFFE) database (Lyons et al., 1998). In a second condition (non-social condition), 20 participants (10 females) received non-social feedback in the form of a tick (also known as a check mark) or an X mark in greyscale.

Participants filled out a debriefing questionnaire after they completed the task.

#### Results and discussion

All data analyses in this and the following experiments were performed off-line using commercial software packages SPSS 22 (IBM Corp., 2013) and MATLAB 8.1 (MathWorks Inc., 2013).

The results of the personality questionnaire showed that all participants displayed normal capacity for recognition of facial expressions. On average, they also presented normal levels of altruism, empathy and attitudes toward reward and punishment (**Table 2**).

**Table 2:** Personality questionnaires average scores for social (Soc.) and non-social (Non-Soc.) groups.

	Faces	Altruism	Drive	FS	RR	BI	EQ	SR	SP
Soc.	17.9 (1.98)	55.3 (10.32)	10.3 (1.62)	11.9 (3.00)	15.7 (2.38)	21.3 (3.49)	39.0 (14.97)	10.9 (3.91)	11.4 (5.36)
Non-Soc.	18.2 (1.09)	56.1 (9.01)	10.9 (1.92)	11.6 (2.08)	17.5 (2.14)	21.4 (2.85)	45.7 (7.92)	9.9 (3.71)	10.2 (4.35)
*p*-value	0.548	0.796	0.263	0.796	0.019	0.866	0.092	0.417	0.454

Table entries indicate average scores and standard deviations (in brackets) for “Reading the Mind in the Eyes” (Faces), “Self Report Altruism,” “Behavioral Inhibition/Approach” questionnaire’s fun seeking (FS), reward responsiveness (RR), behavioral inhibition (BI) components, “Empathy Quotient” (EQ), and “Sensitivity to Punishment and Sensitivity to Reward Questionnaire’s” sensitivity to reward (SR) and sensitivity to punishment (SP) components. One-way ANOVA *p*-values are reported in the last row. Even though reward responsiveness was significantly higher in the non-social group, such a difference between groups would only diminish the effect size.

Results from the debriefing questionnaires indicated that all participants recognized the symbols used in the non-social condition as the notation respectively for positive and negative feedback. One participant reported not having understood the task so his data was excluded from further analysis.

Changes in tipping behavior for the two conditions during the task were tested using the multiple analysis of covariance (MANCOVA) procedure, with tipping percentages in each block (three blocks) as dependent variables, group (two groups) as a between subjects factor, and trial number (1–40) as a covariate. The analysis was run on data concerning the decisions of 39 participants (20 and 19 participants for social and non-social conditions respectively) in the two groups.

Social feedback was found to have a different impact on tipping and learning than non-social reward. Specifically, social feedback was found to have a significant effect on the percentages tipped in blocks 2 and 3 (**Table 3**). Furthermore, considering the whole task, the difference between the social norm and the amount tipped by participants in the social condition was significantly lower than the difference between the social norm and the amount tipped by participants in the non-social condition, i.e., the social group tipped more by 1, 4.9 and 8.2% in blocks 1, 2 and 3 respectively. The first finding confirms the hypothesis that social feedback in the form of facial expressions led participants to display higher degree of generosity, viz. tipping contribution. The second finding confirms that learning is facilitated by social feedback.

**Table 3:** Multiple analysis of covariance (MANCOVA) results for Experiment 1.

Source	Dependent variable	Grand mean (%)	Standard deviation (%)	DoF	*F*	Significance	$\eta_{p}^{2}$
*Group* (IV)	Block 1	14.8	12.3	1	2.526	0.112	0.002
	Block 2	22.9	21.9	1	20.530	0	0.013
	Block 3	23.3	68.1	1	5.653	0.018	0.004
Error	Block 1			1557			
	Block 2			1557			
	Block 3			1557			

Testing was performed on the effects of the independent variable (IV) Group (i.e., the type of feedback received by participants) on the percentage of the bill tipped by participants. The mean tipping percentage (i.e., percentage of the bill tipped) and standard deviation for each block is presented with corresponding degrees of freedom (DoF), *F* statistics, significance (*p*-value), and effect size ($\eta_{p}^{2}$).

A large inter-individual variability of performance was observed, with some participants learning to adapt their decisions to conform to the norm much better than other participants, who seemed to behave independently of the feedback throughout the experiment. Nonetheless, more participants displayed learning in the social condition than participants in the non-social condition. To quantify this, and identify the number of learners in the task, we assumed that if the mean amount tipped by a participant was significantly different from one block to the next and moved toward the underlying social norm, then that participant displayed learning. According to this criterion, we found that 12/20 displayed learning in the social group, vs. 7/19 in the non-social group.

With respect to the scores of the personality questionnaires we administered, no significant difference between groups was found (see **Table 2**). This finding mitigates the concern that the behavioral effects observed could be explained solely by some stable personality trait—for instance, by some autistic trait.

**FIGURE 1:**
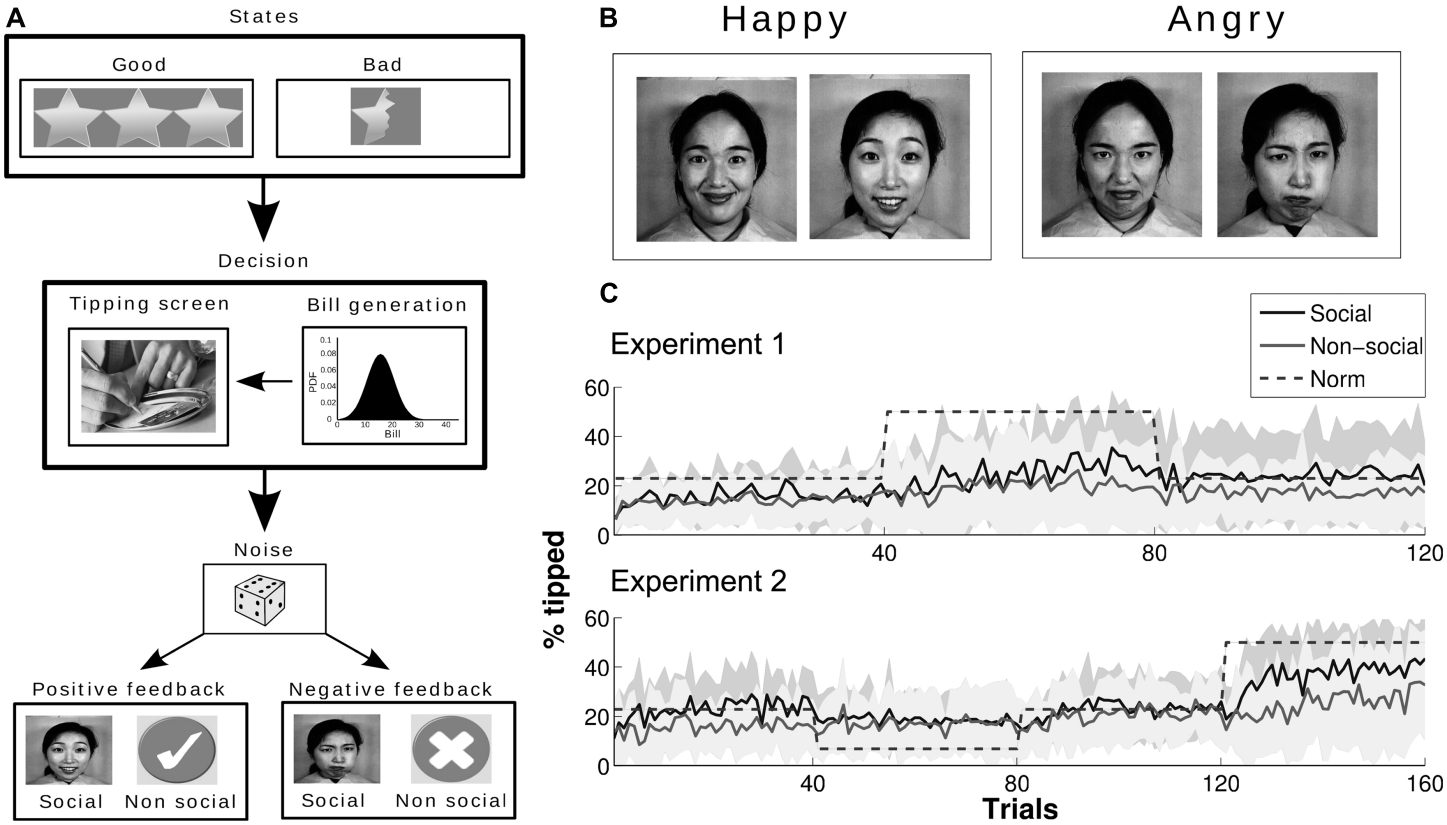
**(A)** Structure of a single trial. First, state of service is presented (either good or bad). Then, the participant is presented with a bill drawn from a normal distribution with μ = 18, σ = 5 (truncated to be >3 and <45). A decision is made, and the participant is presented with either positive or negative feedback (with a certain probability; see **Tables 1** and **4** for details) represented as a face or a tick/cross for social and non-social conditions respectively. The task was implemented using the Matlab Psychophysics Toolbox extensions. **(B)** Faces used as social feedback cues (Lyons et al., 1998). **(C)** Group averaged tipping performances in Experiment 1 (top) and Experiment 2 (bottom). Shaded areas are ±standard deviation; the dashed line is the social norm of tipping.

Two further points should be noted. First, although the difference between the social norm and the amount tipped by participants in the social condition was significantly lower than that for participants in the non-social condition, participants in both groups always tipped less than the norm in the first two blocks. One hypothesis that could explain this finding is that participants had a strong prior bias toward a specific action different from the social norms in our task. Based on questionnaire results, it is plausible that participants, who all lived in the UK for 1 year or more, came to the Tipping Game with a prior of 13%, which would be consistent with the behavior they displayed throughout the experiment. Second, standard deviations from average percentages tipped in both groups were high, especially in the third block (see **Figure 1**). Considering the whole experiment, the standard deviation from the average percentage tipped by the social group was 5.64 vs. 3.39 mu for the non-social group. So, in general, we observed high variability in the behavioral data; in particular, participants in the social group took actions that were spread out over a larger range of values in comparison to the actions taken by participants in the non-social group.

## EXPERIMENT 2

Results from Experiment 1 raised two questions. First, does social feedback impact both learning performance and willingness to be generous to the same degree, or rather one of these two factors more than the other? Experiment 1 could not answer this question because the norms of tipping to be learned were above standard real-life levels (i.e., around 15%). So, learning performance aligned with generosity. If social feedback boosts generosity more than learning, then it will lead to a resistance toward learning a new norm when this norm is lower than the standard; that is, learning performance will be biased toward over-tipping.

Second, does social feedback impact learning performance only when the difficulty of the task is higher? Results from Experiment 1 could be explained simply as a function of the difficulty of the task, namely: the more unreliable was the feedback, the more significant was the difference we found between social and non-social group. If social feedback is effective only when the task is difficult, then no significant difference will be found between groups when the feedback provided is more reliable. Experiment 2 was designed to test these two predictions.

### METHODS

#### Participants

A novel sample of forty participants (12 males) made decisions in the Tipping Game. All participants were students in the University of Edinburgh, signed informed consent approved by the University of Edinburgh Ethics Committee and were compensated with £6/h for taking part in the experiment. While some of the participants were international students, they all lived in the UK for 1 year or more.

#### Design and procedure

This second experiment was different from Experiment 1 in three respects. To test our first prediction, the social norm of tipping in the second block was lower than the standard: it was 7%. To test our second prediction, we added a fourth block, where the reliability of the feedback was higher (see **Table 4**). Including a fourth block, the task in this second experiment was longer than in Experiment 1. So, in order to prevent participants from getting excessively bored or fatigued, we decided not to administer personality questionnaires.

**Table 4:** Feedback structure in Experiment 2.

Probability of positive feedback (tip < norm/tip ≥ norm)	Block 1 (norm 23%)	Block 2 (norm 7%)	Block 3 (norm 23%)	Block 4 (norm 50%)
Good state	20/80	20/80	35/65	5/95
Bad state	30/70	30/70	40/60	10/90

Probability of obtaining a positive feedback as a function of the action that was made (tip < norm/tip ≥ norm), and in the situation where the service was either good (first line) or bad (second line).

#### Results and discussion

Results from the debriefing questionnaires indicated that all participants recognized the symbols used in the non-social condition as the notation respectively for positive and negative feedback.

Changes in tipping behavior for the two conditions during the task were tested using the MANCOVA procedure, with tipping percentages in each block (four blocks) as dependent variables, group (two groups) as a between subjects factor, and trial number (1–40) as a covariate. The analysis was run on data concerning the decisions of 40 in the two groups.

Social feedback was found to have a different impact on learning and social decision-making than non-social feedback, thus confirming the results of Experiment 1. Specifically, social feedback cues were found to have a significant effect on percentages tipped in all blocks (see **Table 5**). Furthermore, the difference between the social norm and the amount tipped by participants in the social condition was significantly lower than the difference between the social norm and the amount tipped by participants in the non-social condition, i.e., social group tipped more by 7.2, 1.8, 2.9 and 10.6% in blocks 1 to 4 respectively.

**Table 5:** Multiple analysis of covariance (MANCOVA) results for Experiment 2.

Source	Dependent variable	Grand mean	Standard deviation	DoF	*F*	Significance	$\eta_{p}^{2}$
*Group* (IV)	Block 1	19.8%	16.8%	1	77.006	0	0.046
	Block 2	17.9%	12.6%	1	8.106	0.004	0.005
	Block 3	21.9%	17.6%	1	10.810	0.001	0.007
	Block 4	31.2%	20.7%	1	117.125	0.000	0.068
Error	Block 1			1597			
	Block 2			1597			
	Block 3			1597			
	Block 4			1597			

*Testing was performed on the effects of the independent variable (IV) Group (i.e., the type of feedback received by participants) on the percentage of the bill tipped by participants. The mean tipping percentage (i.e., percentage of the bill tipped) and standard deviation for each block is presented with corresponding degrees of freedom (DoF), *F* statistics, significance (*p*-value), and effect size (η_p_^2^). Significant differences between percentages tipped by each group were observed in all blocks.*

So, as the percentages tipped by the social group were found to be higher than those of the non-social group across all blocks, the hypothesis receives some confirmation that social feedback in the form of facial expressions led participants to display higher degrees of generosity. As in experiment 1, the second difference we found indicates that social cues may facilitate learning of a social norm. Although in block 2 the social group did not learn the norm better than the non-social group, which suggests that the learning effect observed in all other conditions might be driven by generosity, more participants displayed learning in the social condition than in the non-social condition. According to the learning criterion described above, we found that 17/20 displayed learning in the social group, vs. 10/20 in the non-social group.

## A MODEL OF THE TIPPING GAME

To further describe quantitatively the nature of the effects that we observed, we used a Rescorla–Wagner reinforcement learning algorithm (Rescorla and Wagner, 1972) to model the behavior of participants in our two experiments. The model algorithm makes decisions in our task with the goal of maximizing its total reward. It does this by learning action values *Q* for state-action pairs, and selecting actions in function of their estimated *Q*-values at each trial.

The possible states were two, corresponding to “good” or “bad” service quality. The action space comprised 101 actions, corresponding to tip percentages from 0% increasing in steps of 1 to 100%. For each of the two states, the action taken by the model was iteratively assigned a value *Q*, which was a function of the reward obtained for taking that action (a combination of feedback received and economic cost incurred, see below) and the *Q*-value of that state-action pair stored in memory. This is expressed by the *Q*-update equation:

(1) $$Q{\left(state,action\right)}_{new}=Q{\left(state,action\right)}_{old}+\alpha \left(reward-Q{\left(state,action\right)}_{old}\right)\,$$

where α is the learning rate (0 ≤ α ≤ 1). The smaller α, the less the existing knowledge is modified. Conversely, as α tends to 1, what has already been learned can be quickly overwritten. The reward signal in our model consisted of the hyperbolic tangent (scaling to interval [-1 1]) of the weighted difference of two components: an economic component and a reward outcome component. Formally:

(2) Reward=tanh⁡(woutrout−weconrecon)

where *r*_econ_ is an economic factor that is equal to the Tip/Bill ratio. As we assumed that the model could tip at most an amount equal to the bill, the range of this economic factor is 0 ≤*r*_econ_ ≤ 1. The economic weight *w*_econ_ is a parameter that determines the extent to which spending money in tips was valued in the Tipping Game. We assumed that, *at best*, spending money in tips would not have any negative impact on an agent’s reward signal, and we set the range of this parameter to 0 ≤*w*_econ_ ≤*w*_max_, where *w*_max_ is 10. So, lower values of *w*_econ_ indicate that the agent does *not* mind spending money in tips. Conversely, higher values of *w*_econ_ indicate that the agent does mind spending money in tips. If the agent does not mind spending money in tips at all (viz., *w*_econ_ = 0), then the economic cost of a tip, whatever its amount, will not weigh against the agent’s overall reward. So, if the agent displays *w*_econ_ = 0, then it will systematically over-tip in the Tipping Game. Roughly, lower values of *w*_econ_ correspond to a more nonchalant attitude toward tipping—whatever the amount. The reward outcome factor *r*_out_ is associated to the two possible outcomes in the task: either positive feedback or negative feedback. The reward magnitude of positive feedback is assumed to be 1, while the magnitude of negative feedback is assumed to be 0. The outcome weight *w*_out_ (0 ≤*w*_out_ ≤ *w*_max_) is a parameter that determines to what extent positive feedback was valued. Higher values of *w*_out_ correspond to a more positive feedback-seeking attitude. When *w*_econ_ > *w*_out_, it should be expected that the agent will severely under-tip in the Tipping Game. When *w*_econ_ < *w*_out_, the learning behavior of the agent in the Tipping Game will depend heavily on the action selection mechanism, in particular on how the exploration of the action space is performed.

Finally, the model made choices according to the “softmax” rule:

(3) $$p\left(action|state\right)=\frac{\exp\left(\tau Q\left(state,\;action\right)\right)}{\overset{N}{\underset{i=1}{\Sigma }}\exp\left(\tau Q\left({state,\;action}_{i}\right)\right)}$$

which determines the probability of selecting a certain action (e.g., a tip of 11% of the bill) for a given state (e.g., “good” service). The parameter τ featuring in (3) is positive and is called “inverse temperature.” A low τ causes the actions to be all nearly equiprobable; whereas, as τ gets larger, the action with the highest expected reward has a much higher probability of being selected than the other actions.

### MODELING RESULTS

To estimate parameters values, we fitted the model to each participant using maximum likelihood, resulting in a set of parameters (i.e., α, τ, *w*_out_ and *w*_econ_) that maximized the probability that the model-agent would make the same choices as the participant (on average over the course of the experiment).

The parameter values obtained confirmed that participants in the social group displayed different learning and decision-making profiles from participants in the non-social group. For the two experiments, a large difference between the social and non-social group was found in the economic weight *w*_econ_: in comparison to participants in the non-social condition, participants in the social condition were much less sensitive to economic costs, thereby displaying a more generous tipping behavior (**Table 6**). This difference is partly explained by the number of learners in each group. Smaller, but non-zero, values of *w*_econ_ were characteristic of participants who displayed a better learning performance, and, according to the learning criterion we employed (see Results and Discussion), the number of learners in the social group was larger than the number of learners in the non-social group. So, it should be expected that the larger the difference in the number of learners between the two groups, the larger is the difference between the economic weights *w*_econ_ that characterize the two group’s tipping behavior.

**Table 6:** Average model parameters for the two experiments.

	Group	α	*T*	*w*_out_	*w*_econ_
Experiment 1	Social	0.24	93.2	4.43	0.004
	Non-social	0.2	78.8	3.82	0.038
Experiment 2	Social	0.4	20.7	5.68	0.002
	Non-social	0.29	59.9	4.51	0.081

Significant differences (*p* = 0.019 and *p* = 0.049 in Experiments 1 and 2 respectively; independent two-tailed *t*-tests) were found in economic weight values between the groups (*w*_econ_).

## GENERAL DISCUSSION

Our study asked how the type of feedback obtained by people after they make decisions in social situations affect the way they learn a social norm. We addressed this question by determining whether the influence of facial expressions on participants’ decisions in a novel associative learning task called the “Tipping Game” was significantly different from the influence of non-social feedback in the form of conventional marks. We found that participants receiving feedback in the form of happy or angry facial expressions behaved in a significantly different way than participants receiving feedback in the form of tick or cross marks. This effect was observed across most blocks in our task, and, specifically, had impact on how much participants were willing to give as a tip and on how well they learned the underlying social norm. We must however note that the observed effect sizes were small (cf., **Tables 3** and **5**).

In order to explore quantitatively our participants’ behavior, we used a version of the Rescorla–Wagner algorithm to model performance in the Tipping Game. Modeling results show that differences in the attitude toward economic costs, captured by *w*_econ_, could account for the behavioral differences much better than differences in outcome weights, rate of learning or action selection strategy.

Taken together, our findings confirm the potent role of social reinforcements on learning, which is predicted by classical social learning and facilitation theories (Zajonc, 1965; Bandura, 1977; see also Doris and Nichols, 2012). Consistent with previous findings (e.g., Hurlemann et al., 2010), social feedback in the form of facial expressions, if compared to non-social feedback in the form of conventional feedback marks, led people to be more generous. Furthermore, both behavioral and modeling results confirmed that social reward in the form of facial expressions bias decision-making, which is consistent with previous behavioral and modeling results focused on smiling facial expressions (Averbeck and Duchaine, 2009; Shore and Heerey, 2011). Specifically, our results extend the literature on the impact of subtle cues on social behavior (e.g., Bargh and Williams, 2006) indicating that emotional facial expression may bias behavior toward more generosity through lowering one’s sensitivity to economic costs.

Angry facial expressions, in particular, might drive such a pattern of learning and decision-making by affecting the motivation to spend extra monetary units during a social interaction, thereby displaying more generous behavior. Angry facial expressions might signal social disapproval of a failure to comply with a norm. Such a failure might be due to a lack of knowledge of the social environment. Thus, learners of a social norm might feel anxious and uncomfortable in observing an angry reaction, which might in turn lead them to behave more generously in an attempt to avoid social disapproval in the future (Fehr and Gächter, 2002; Haley and Fessler, 2005; Bateson et al., 2006; Rigdon et al., 2009). Interestingly, the desire to avoid social disapproval is one of the main factors that apparently motivate people to tip in restaurants in the real world (Conlin et al., 2003; Azar, 2007).

Given the structure of the Tipping Game, and given that our Rescorla–Wagner model nicely fitted participants’ choices, it is plausible that our task engaged a basic reinforcement learning mechanism, which is responsible for several social behaviors (Frith and Frith, 2012). According to reinforcement learning models (Sutton and Barto, 1998), estimated value of states or actions is updated through prediction error signals, whether about primary reward, money, or conventional or social choice feedback. These error signals have been found to accurately predict neural responses in the mesostriatal dopaminergic system and its targets (Montague et al., 1996; Niv, 2009; Colombo, 2014). Some findings suggest that both social and non-social reward are processed by the same striatal neural systems (Lin et al., 2012a), while other studies emphasize the specific role of mentalizing networks, associated with the temporoparietal junction, in processing social reward (e.g., Evans et al., 2011). In light of these results, one question for further research is whether a domain general, dopamine-based reinforcement learning mechanism can be sufficient for the acquisition of social norms (Ruff and Fehr, 2014). Examining results from the Tipping Game task using functional magnetic resonance imaging (fMRI) can be one way to make progress on that question.

Three limitations of our study should be singled out. First, some neurodevelopmental disorders, including autism, Asperger syndrome and schizophrenia, are known to affect the relative power of social vs. non-social feedback (cf., Montague et al., 2012). Furthermore, previous experiments suggest that males and females process some types of social reward differently (Spreckelmeyer et al., 2009). While we measured some scores relevant to assess autistic traits in Experiment 1, we did not collect them in Experiment 2, and we did not measure other personality traits potentially relevant to social decision-making and learning. Based on the scores that we did collect, and based on analyses of gender differences, the hypotheses that the behavioral effects we found could be explained solely by a gender difference or by some autistic trait in our participants could be ruled out. However, the issues remain to what extent gender differences in reward processing are associated with differences in social learning and decision-making, and how the social dysfunctions observed in people with certain neurodevelopmental disorders is linked to a specific deficit in the processing of social feedback.

Second, one reason why our participants did not generally perform well in the Tipping Game might be that its feedback structure made the learning task especially hard. Overall, the level of noise in the mapping between state-action pairs and reward outcomes was high across the blocks in our task, making the feedback provided not very reliable. Moreover, the reliability of the feedback was independent from the difference between amount tipped and underlying social norm, so that tips well above the social norm could still receive negative feedback. In order to improve learning performance, the feedback structure of the task may be modified in two ways. On the one hand, the level of noise in the mapping between state-action pairs and reward outcomes may be diminished across all blocks, similarly to what we did in the fourth block of Experiment 2. On the other hand, the reliability of the feedback provided may be made dependent on the distance between amount tipped and underlying social norm, so as to strengthen the reliability of feedback for tips well above or well below the social norm. Although our main behavioral results were robust to different levels of noise, it remains a question for further research how exactly social decision-making and learning are affected by the uncertainty of feedback information.

The third limitation concerns the distinction between social and emotional cues. The stimuli that we used in the social condition of our task did not help us to determine whether the behavioral effects we observed depended on social rather than on only the emotional dimension of facial expressions. Facial expressions convey both social and emotional information. Besides communicating information about other agents, facial expressions can often elicit emotional reactions in the observers (Furl et al., 2012). In order to identify the role of emotional cues alone, in contrast to facial expressions, on participants’ learning and social decision-making, a third condition for our task may employ emotional, non-social reward. Further work will aim at clarifying these issues, and the generality of our findings.

## Conflict of Interest Statement

The authors declare that the research was conducted in the absence of any commercial or financial relationships that could be construed as a potential conflict of interest.
